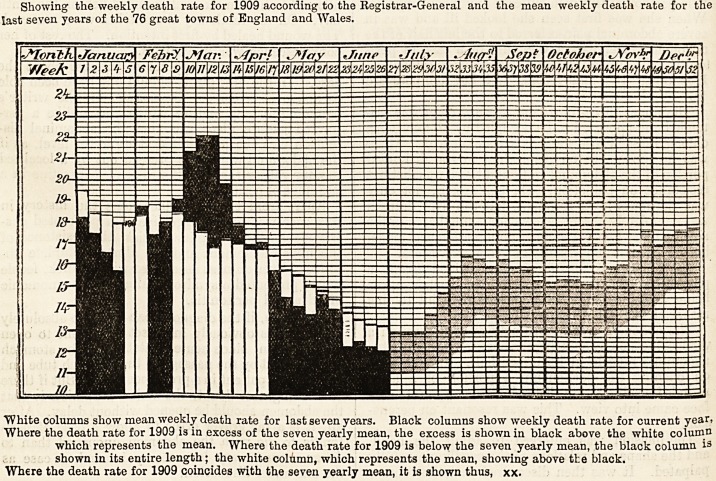# Diagram of the Weekly Death Rate in 1909

**Published:** 1909-07-10

**Authors:** 


### Public Health and Hygiene.

### DIAGRAM OF THE WEEKLY DEATH RATE IN 1909.

Showing the weekly death rate for 1909 according to the Registrar-General and the mean weekly death rate for the last seven years of the 76 great towns of England and Wales.

White columns show mean weekly death rate for last seven years. Black columns show weekly death rate for current year. Where the death rate for 1909 is in excess of the seven yearly mean, the excess is shown in black above the white column which represents the mean. Where the death rate for 1909 is below the seven yearly mean, the black column is shown in its entire length; the white column, which represents the mean, showing above the black.

Where the death rate for 1909 coincides with the seven yearly mean, it is shown thus, xx.

## Figures and Tables

**Figure f1:**